# Import volumes and biosecurity interventions shape the arrival rate of fungal pathogens

**DOI:** 10.1371/journal.pbio.2006025

**Published:** 2018-05-31

**Authors:** Benjamin A. Sikes, Jennifer L. Bufford, Philip E. Hulme, Jerry A. Cooper, Peter R. Johnston, Richard P. Duncan

**Affiliations:** 1 Department of Ecology and Evolutionary Biology and Kansas Biological Survey, University of Kansas, Lawrence, Kansas, United States of America; 2 Bio-Protection Research Centre, Lincoln University, Lincoln, New Zealand; 3 Landcare Research, Lincoln, New Zealand; 4 Landcare Research, Auckland, New Zealand; 5 Institute for Applied Ecology, University of Canberra, Canberra, Australian Capital Territory, Australia; University of York, United Kingdom of Great Britain and Northern Ireland

## Abstract

Global trade and the movement of people accelerate biological invasions by spreading species worldwide. Biosecurity measures seek to allow trade and passenger movements while preventing incursions that could lead to the establishment of unwanted pests, pathogens, and weeds. However, few data exist to evaluate whether changes in trade volumes, passenger arrivals, and biosecurity measures have altered rates of establishment of nonnative species over time. This is particularly true for pathogens, which pose significant risks to animal and plant health and are consequently a major focus of biosecurity efforts but are difficult to detect. Here, we use a database of all known plant pathogen associations recorded in New Zealand to estimate the rate at which new fungal pathogens arrived and established on 131 economically important plant species over the last 133 years. We show that the annual arrival rate of new fungal pathogens increased from 1880 to about 1980 in parallel with increasing import trade volume but subsequently stabilised despite continued rapid growth in import trade and recent rapid increases in international passenger arrivals. Nevertheless, while pathogen arrival rates for crop and pasture species have declined in recent decades, arrival rates have increased for forestry and fruit tree species. These contrasting trends between production sectors reflect differences in biosecurity effort and suggest that targeted biosecurity can slow pathogen arrival and establishment despite increasing trade and international movement of people.

## Author summary

When people and goods move around the world, they spread nonnative species—including pathogens that can cause disease—leading to huge economic impacts. Many countries try to limit pathogen arrivals by screening goods and people before they enter. But are these biosecurity measures effective? Pathogens are hard to detect, and we rarely have data on key metrics such as the volume of goods imported, number of people arriving, and new nonnative pathogens establishing over time. Our study uses a database of all known New Zealand plant pathogen records to estimate how many fungal pathogens arrived and established on 131 economically important plant species each year over the last 133 years. Pathogen arrivals increased exponentially for 100 years starting in 1880, paralleling an increasing volume of goods imported. Since about 1980, the rate of new pathogen arrivals has stopped increasing, despite imports and the arrival of people continuing to accelerate. However, these recent trends differ among plants from different economic sectors. Pathogen arrivals on crop and forage plants have declined but continue to increase on forestry and fruit trees. This trend reflects differences in the biosecurity measures imposed, suggesting that targeted biosecurity can reduce the establishment of nonnative pathogens even while global trade and travel continue to increase.

## Introduction

International movements of goods and people are major pathways for transporting species to new regions and can result in harmful biological invasions [1,2]. Over the last half century, international trade and travel have risen dramatically [3,4] in parallel with large increases in the arrival and establishment of nonnative species [5–9]. Worldwide, the number of nonnative species in different regions of the world correlates with the magnitude of trade imports in a range of taxa [10–12], and within regions, trade measures are closely linked to new species’ arrival and establishment rates [10,13]. International travellers also transport nonnative species, including plants, pathogens, and invertebrates, some of which establish as biological invaders [14–16]. Forecasts predict continued increases in international trade and travel and more links among countries [17,18]. Based on historical patterns, these increases have the potential to accelerate the arrival and establishment of nonnative species in new regions [19,20], with consequent economic and ecological impacts.

To counter the threat posed by the arrival of unwanted species through trade and transport pathways, many developed countries have invested heavily in border biosecurity surveillance [21], phytosanitary inspection, and quarantine. Biosecurity measures are designed to prevent unwanted or unknown species entering trade or transport pathways, to detect species arriving in trade shipments or with passengers, and to prevent the release of species into the wild [22–26]. Effective biosecurity is particularly important to countries that rely heavily on primary production, because new pests and diseases that threaten plant or animal health can have major economic consequences [7,23]. Developed countries have invested more in biosecurity than less developed nations [27–30], but even developed countries have difficulty assessing the value of their biosecurity investment because costs are often spread across multiple agencies, and the benefits of such interventions are often unclear [31]. New Zealand is a major exporter of primary produce and one of the few countries that provide a nationwide accounting of biosecurity investment, spending more than US$137 million in 2014 [32], slightly more than 0.3% of its gross domestic product (GDP). Justifying this substantial expense requires that biosecurity measures cost less than the economic and ecological costs of the pest, pathogen, and weed incursions that are prevented by such interventions [33,34].

Pathogens are key biosecurity targets because they can readily enter transport pathways and pose significant threats to animal and plant health [7,11,35]. Plant fungal pathogens are responsible for crop yield losses that cost individual economies billions of dollars annually [7,36,37], with impacts across a wide range of production sectors, including agriculture, forestry, horticulture, and livestock [7,38,39]. Despite substantial investment by countries in biosecurity and global initiatives to coordinate these efforts [40,41], it has proven difficult to evaluate the effectiveness of biosecurity measures. Quantifying how pathogen arrival and establishment rates have changed over time is especially problematic because pathogens are difficult to detect in the early stages of invasion. Extensive host surveys are often necessary for initial detection and require expert pathologists to isolate and identify pathogens in symptomatic hosts. These difficulties make detecting new pathogens particularly sensitive to variation in survey effort [42,43].

Here, we use a long-term, comprehensive database of all known associations between nonnative plants and fungal pathogens in New Zealand [44] to examine trends in fungal pathogen arrival rates over time in relation to changing trade and transport patterns while accounting for variation in sampling effort. We use these data to evaluate whether biosecurity investment has been effective in reducing pathogen establishment. We focus on nonnative host plants in 4 primary production sectors that are major targets for biosecurity in New Zealand: crops (46 species, including wheat, tomatoes, and onions), fruit trees (30 species, including grapes, apples, and kiwifruit), commercial forestry (42 species, including pines and eucalypts), and pastures (13 species of forage grasses and legumes). Because these are the major primary production sectors, plant species in these groups comprise the majority of host–pathogen records for well-sampled species in New Zealand. We use these data to estimate how annual rates of pathogen arrival have changed over time while accounting for variation in survey effort and then to address three questions: (1) Is the overall rate of nonnative fungal pathogen establishment in New Zealand more strongly linked to changes in import trade volume or passenger arrivals? (2) Do changes in pathogen arrival rates differ among the primary production sectors, and are changes related to variation in sector-specific imports? (3) Do changes in pathogen arrival rates over time coincide with the implementation of specific biosecurity measures?

## Results and discussion

**Fig 1:**
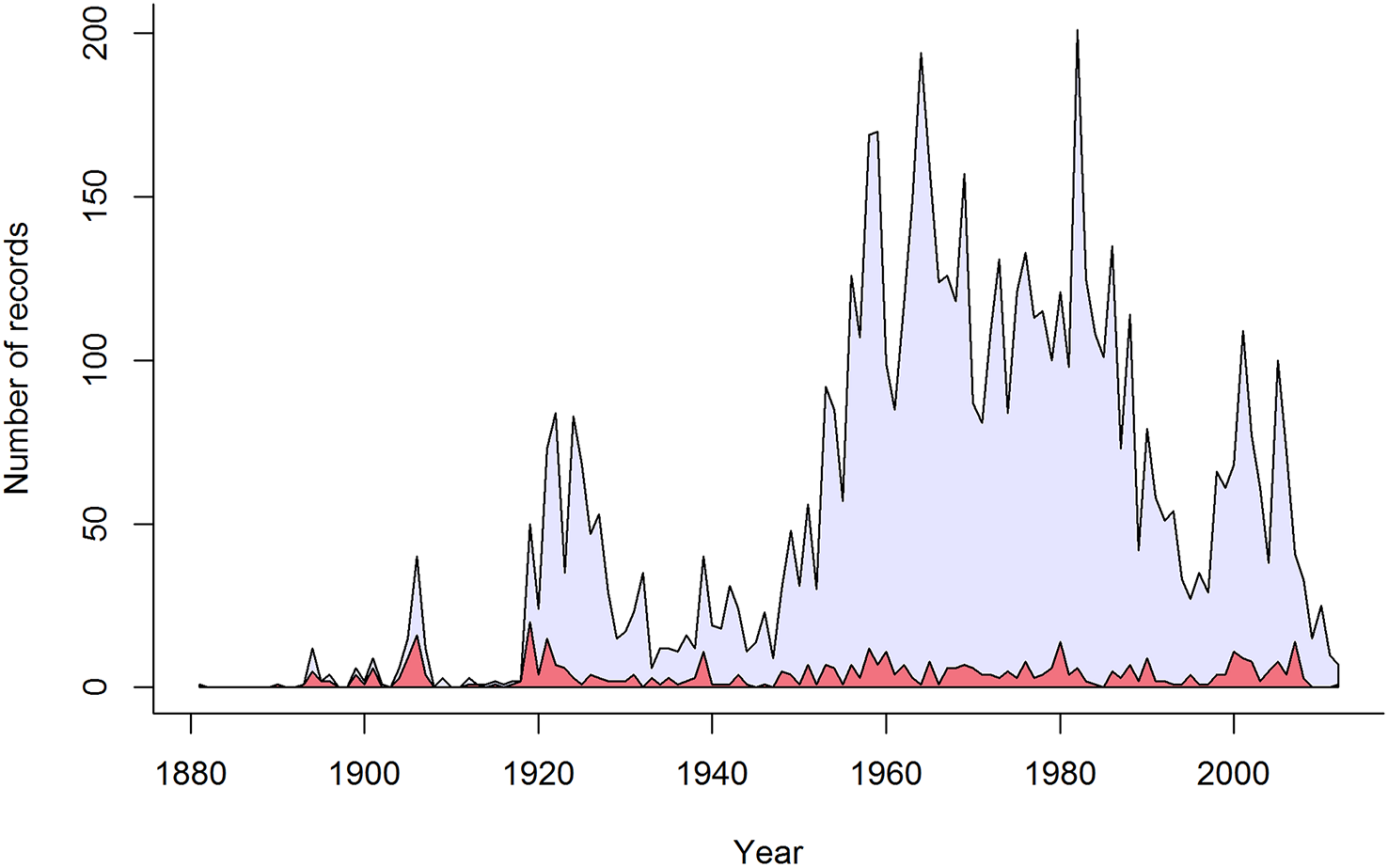
The number of host–pathogen records from New Zealand for our focal host plants from 1881–2012. Light blue is the total number of host–pathogen records per year, which we used as a measure of sampling effort (*N*_*t*_), and red is the number of new pathogen species discovered each year (i.e., the first record of a nonnative pathogen species in New Zealand on one of the focal host plants).

**Fig 2:**
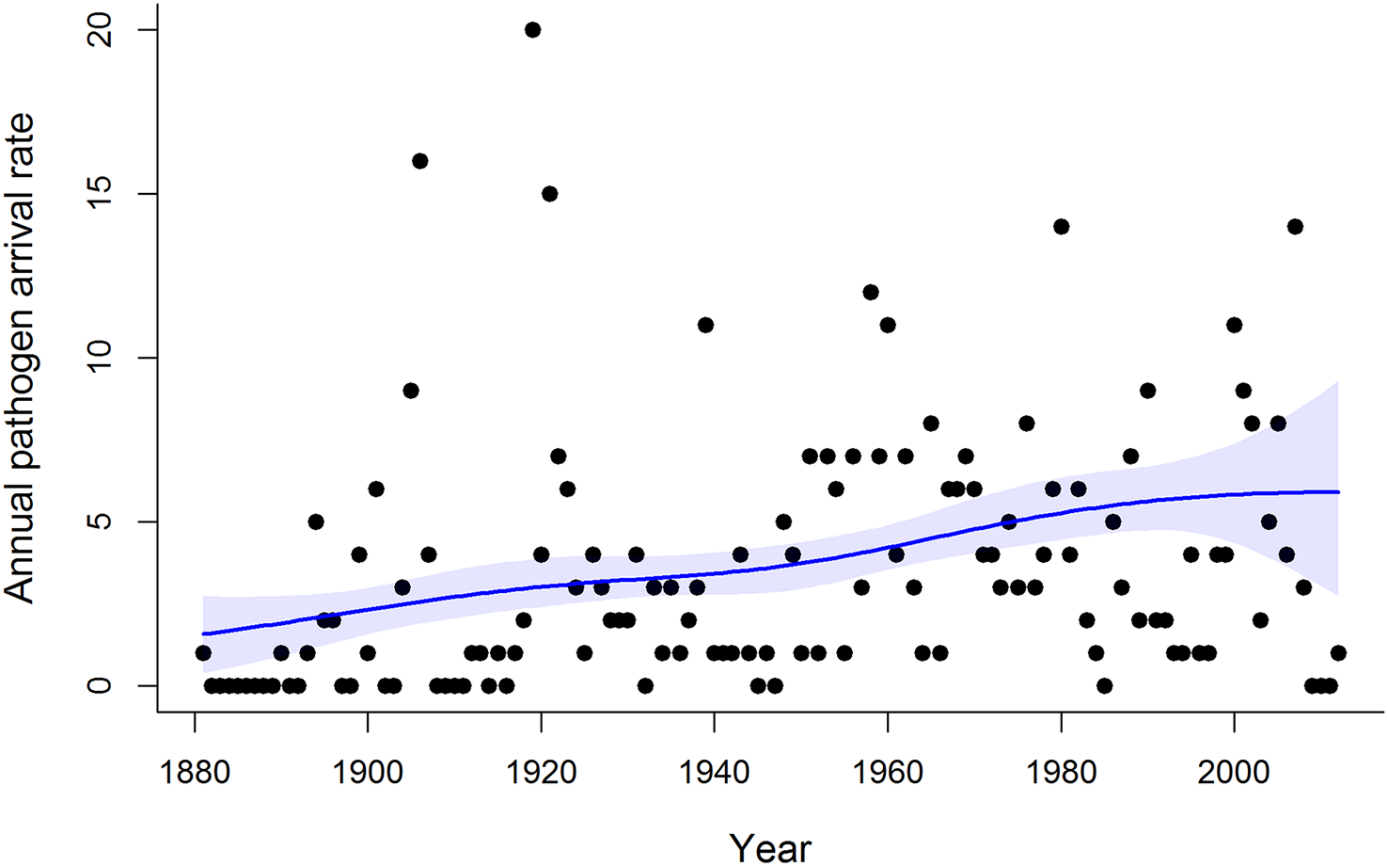
The estimated average annual arrival rate for fungal pathogens. The number of new pathogens discovered each year on 131 focal host plant species in New Zealand (closed circles) and the mean annual rate of pathogen arrival estimated from the model (solid blue line), with shading showing the 95% credible interval.

The data comprised 6,691 host–pathogen records from 131 nonnative host plant species spanning the years 1881–2012. We restricted our analysis to the 466 pathogen species whose first New Zealand record was on one of the 131 focal host plants, which identified these hosts as the source of the new pathogen arrivals. Time series plots revealed substantial variation in the number of host–pathogen records per year (Fig 1), indicating substantial variation in annual survey effort, which we accounted for in our analyses (Materials and methods). The estimated annual rate at which new fungal pathogens arrived and established on the focal host plants increased from the 1880s until about 1980, after which the annual arrival rate slowed, albeit with wide uncertainty around recent arrival rates (Fig 2). Since 2000, we estimate an average of 5.9 new species of fungal pathogens per year have established on the focal host plant species.

**Fig 3:**
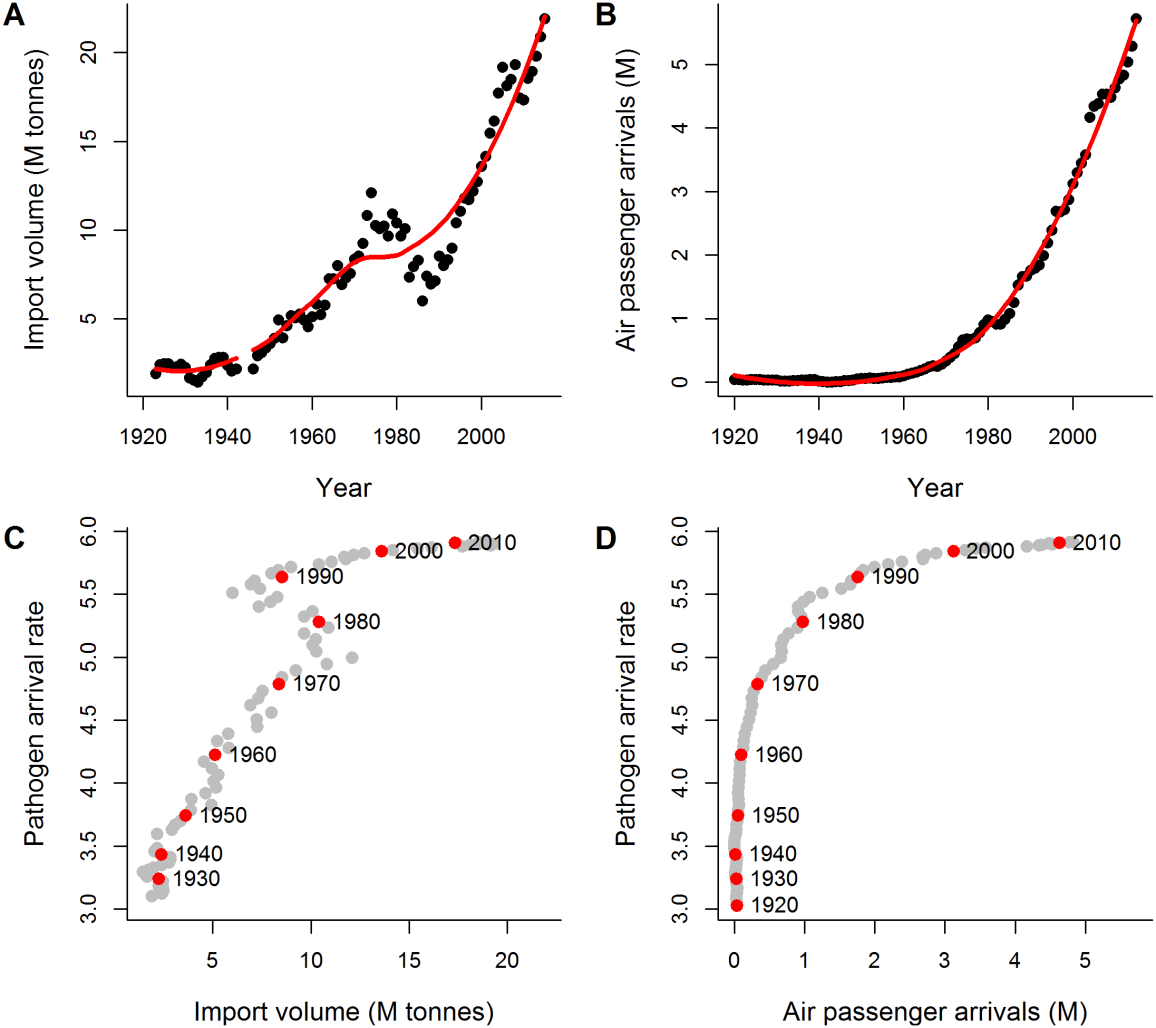
Import volume, passengers arriving, and their relation to annual rate of fungal pathogens arriving in New Zealand. (A) Annual volume of trade imports over time as yearly imports (in M of tonnes; closed circles) and the time-averaged trend (solid red line, obtained by fitting a loess smoothing function); (B) Annual air passenger arrivals to New Zealand over time as yearly arrivals (in M of travellers; closed circles) and the time-averaged trend (solid red line, obtained by fitting a loess smoothing function); (C) Mean annual rate of pathogen arrival as a function of annual import trade volume and (D) as a function of air passenger arrivals. The start of each decade is indicated with a red circle. M, millions.

In contrast to the slowdown in pathogen arrivals, both import trade volume and passenger arrivals to New Zealand have increased dramatically in recent decades, with import volume starting to accelerate in the 1940s and international passenger arrivals in the 1970s (Fig 3A and 3B). To directly compare changes in trade volume and number of passengers with changes in pathogen arrival rates, we plotted the mean values for these variables in each year (Fig 3C and 3D). These plots indicate that pathogen arrival rates were more strongly linked to import volume than to passenger arrivals, with pathogen arrival increasing in concert with increasing import volume until about 1980, when import volume declined briefly and then increased rapidly while pathogen arrival rates slowed (Fig 3C). In contrast, passenger arrivals changed little between 1920 and 1980, during which time pathogen arrival increased, while the substantial and rapid rise in passenger arrivals since about 1980 coincides with slowing of pathogen arrival rates (Fig 3D). These trends suggest that pathogen arrival into New Zealand was most strongly linked historically to increasing import trade volumes, but this relationship has weakened significantly since about 1980.

**Fig 4:**
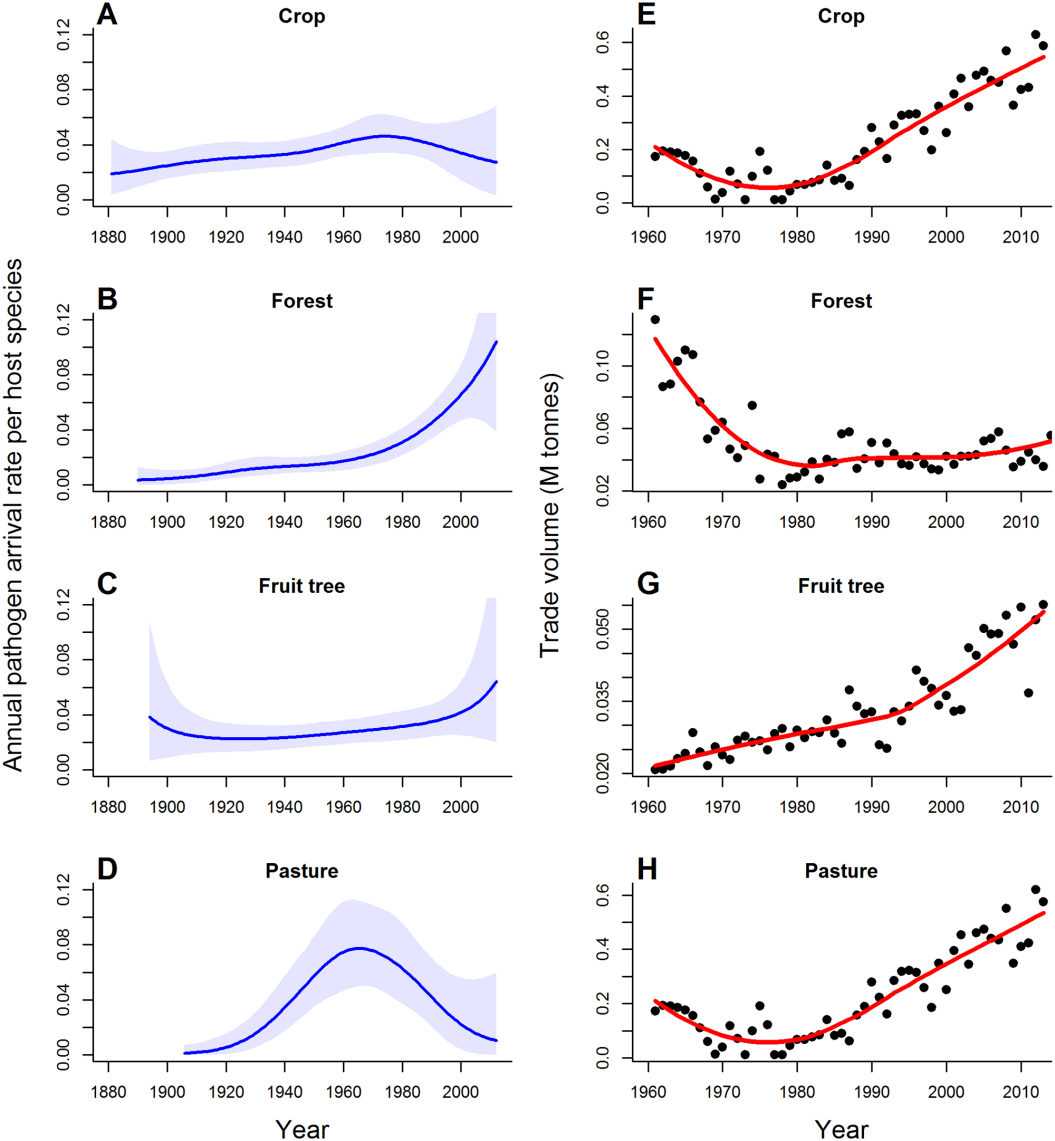
Sector-specific pathogen arrival rates and import volumes. (A–D) Mean annual rate of new pathogen arrival over time for host species in each of the four production sectors (solid line) and 95% credible intervals (shaded); (E–H) Sector-specific import data from 1960–2012 for the corresponding production sector (closed circles) with the time-averaged trend (solid red line, obtained by fitting a loess smoothing function). M tonnes, millions of tonnes.

**Fig 5:**
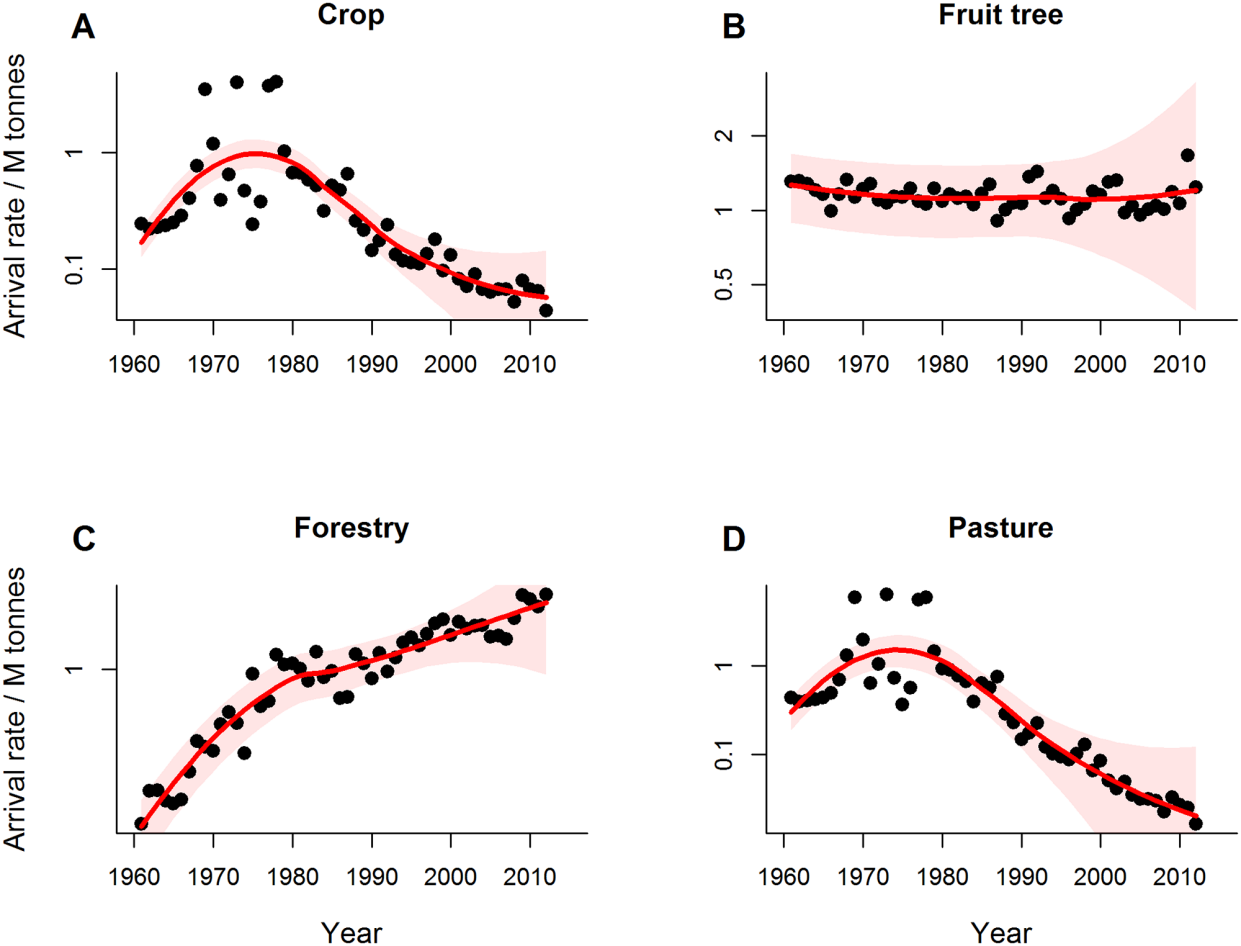
For each production sector, the annual pathogen arrival per species as a function of import volume. Mean annual pathogen arrival rate per species per M tonnes of import volume over time for each of the four production sectors (closed circles) with the trend (solid red line with 95% confidence intervals, obtained by fitting a loess smoothing function). M, million.

Trends in overall pathogen arrival rates, however, obscure substantial variation among the four production sectors. Pathogen arrival rates have declined in recent decades for both pasture and crop species, with declines beginning around the 1970s for crops and slightly earlier for pasture species (Fig 4A and 4D). In contrast, pathogen arrival rates have continued to accelerate for forestry and fruit tree species, especially in recent decades (Fig 4B and 4C). These trends were not consistently associated with changes in sector-specific import volumes since 1960, the period for which sector-specific trade data were available (Fig 4E and 4H; see Materials and methods). Pathogen arrival rates on crop and pasture species have declined since 1960, while import volumes have increased in these sectors. Pathogen arrival rates on forestry species have increased despite declining import volume, while pathogen arrival rates on fruit trees have been relatively steady or increased slightly while trade volume has risen steadily (Fig 4). Consequently, the pathogen arrival rate per host species per million tonnes of import trade has declined for pasture and crop species since about 1980 (Fig 5A and 5D). In contrast, pathogen arrival rate per host species per million tonnes of import trade has remained steady for fruit trees but has increased markedly for forestry species since 1960 (Fig 5B and 5C). Thus, the recent stabilisation in overall pathogen arrival rates (Fig 1) is the sum of contrasting trends among the different production sectors.

Two processes could explain the recent declines in pathogen arrival rates for crop and pasture species despite continued increases in import trade. First, as fungal pathogens arrive, there will be progressively fewer pathogens remaining elsewhere to be introduced [13,20], and pathogens not yet introduced are more likely to be those with a lower probability of transport or establishment [9]. This would imply the pool of readily transported and highly invasive fungal pathogens associated with crop and pasture species is being exhausted, leading to a decline in arrival and establishment rates. While this is a possibility, a recent review found that only about one-third of global pest and pathogen species associated with crops grown in New Zealand were currently present in the country [45]. Moreover, of the ten host plant species with the most fungal pathogens in our data, seven still have fewer than 40% of the fungal pathogens recorded for these species globally [46, See S1 Table]. This implies that a substantial fraction of pathogens have yet to arrive in New Zealand and that saturation is unlikely to explain declining rates of pathogen arrival. This is consistent with models using the distribution of agricultural pathogens coupled with trade patterns to evaluate the risk of new pathogen arrivals, which indicate New Zealand has a moderate to high risk of future plant pathogen invasions [30].

A second explanation for the decline in pathogen arrival rates is increased biosecurity. New Zealand has a long history of plant biosecurity [47]. Yet, consolidated data on government biosecurity spending are only available beginning in the 1990s, and our results show declines in pathogen arrival rates for crop and pasture species commenced much earlier, in the 1960–1970s (Fig 4). Historical developments in plant biosecurity in New Zealand, however, are consistent with the timing of declines in pathogen arrival rates for crop and pasture species. Agricultural biosecurity ramped up in the 1950s, marked by the establishment of the Plant Quarantine Service in 1952 soon after New Zealand signed the International Plant Protection Convention. This is evident in our data, with a substantial increase in pathogen survey effort commencing in the 1950s (Fig 1). A more unified border protection service with a strong legal mandate emerged in 1962 as the Port Agriculture Inspection Service, which evolved further to manage cargo, air, and passenger pathways as the Agriculture Quarantine Service in 1981 [47]. This increase in capacity and effort in agricultural border biosecurity coincides with a weakening relationship between trade and pathogen arrival rates and suggests that biosecurity efforts played a role in limiting new pathogen arrivals.

Biosecurity initiatives targeting pathways specific to pastures and crops are also consistent with the timing of declines in pathogen arrival rates in these sectors. Most pasture and crop species are imported as seed. Voluntary, industry-backed seed certification for agricultural species began as early as the 1920s [48]. However, New Zealand’s entry into the Organisation for Economic Co-operation and Development’s (OECD) seed certification scheme in 1967 likely led to significant improvements in the management of seed-borne diseases, particularly those from overseas [48]. The combination of government investment in agricultural quarantine coupled with an industry-based seed certification scheme targeted key pathways by which pathogens of crop and pasture species entered the country, which could explain the decline in pathogen arrival rates in these sectors from the 1960s onward (Figs 4 and 5).

In contrast, the forestry and fruit tree sectors do not appear to have placed as much emphasis on preborder biosecurity, which could account for the ongoing increase in pathogen arrival in these sectors (Figs 3 and 5). The New Zealand seed certification scheme did not include horticultural species [48], and although phytosanitary inspections of timber imports began in 1949, they focussed primarily on invertebrate pests [47], while broader forestry biosecurity efforts focussed on treating existing tree diseases rather than preventing new arrivals [49,50]. Our data on pathogen survey efforts reinforce these differences among sectors (Table 1): Relative to pasture and crop species, fruit tree and forestry species had, on average, fewer records per species, individual species surveys began later, and peak survey effort occurred several decades later (1960s for pasture and crop species; 1980 and 2000 for fruit tree and forestry species, respectively).

**Table 1:** Summary of plant pathogen records for host plant species in the four production sectors. Columns labelled 95% CI show 95% confidence intervals for the mean value in the previous column.

Sector	No. of species	No. of records	Mean year of first record per species	95% CI	Mean no. of records per species	95% CI	Decade with greatest no. of records
Pasture	13	1,122	1922	1911–1932	86	47–125	1960
Crop	46	2,779	1921	1914–1928	60	42–79	1960
Fruit tree	30	1,589	1930	1918–1941	53	33–73	1980
Forestry	42	1,201	1949	1942–1956	29	18–39	2000

**Abbreviation**: CI, confidence interval; No., number

Pathogens of forestry and fruit tree species have additional potential vectors, including soil and live plant material (e.g., rootstock) and untreated wood products (e.g., wood pallets), that may facilitate further pathogen arrival [51,52]. Postentry quarantine of live plant material, implemented in the 1990s [53], should have slowed arrival rates via this pathway, but no corresponding decrease is evident in pathogen arrival rates (Fig 4B and 4C). This may be because wood packaging, which is used extensively in transporting goods, is potentially a significant pathogen source, and wood packaging volume is likely to have increased in concert with rapidly increasing import trade volumes, potentially contributing to the continued rise in pathogen arrivals for woody species [51,52,54]. International phytosanitary standards for wood products are relatively recent (2002) and are not used for all transport methods, and even treated wood packaging material can still harbour pathogens [54]. Since the relevant trade standard (ISPM-15 dealing with the treatment of wood packaging in international trade; [55]) was only recently revised to include more stringent treatment guidelines, it is likely too early to assess whether this might reduce pathogen arrival rates for woody species.

International travellers can be vectors for nonnative species, and New Zealand has invested heavily in preventing incursions via this pathway using, for example, soft-tissue X-ray machines and detector dogs at international airports since 1996 [56]. Prior to that time, the Ministry for Agriculture and Forestry estimated that it was detecting only 55% of risk goods brought in by passengers, with detection levels rising to 95%–100% after 2001 [56]. These initiatives, however, occurred at least a decade after the observed decline in pathogen arrival rates for crop and pasture species, suggesting that for plant pathogens, other measures were responsible for slowing arrivals.

Postborder pathogen survey efforts to detect new incursions have declined since about 2000 despite the increase in pathogen arrival rates for forestry and fruit tree species (Fig 1). This decline makes it more difficult to evaluate trends in arrival rates, as revealed by the wide uncertainty intervals associated with arrival rate estimates in recent years (Figs 2 and 4). Moreover, there is a time lag between the arrival of new pathogens and their discovery, the length of which will depend on survey effort. We statistically controlled for this in our analysis by explicitly modelling the processes of pathogen arrival and discovery (Materials and methods). This provided an estimate of the number of pathogen species that had arrived and established on the focal host plants but had not yet been detected. In addition to the 466 known pathogen species, we estimated a further 55 species (95% credible interval 30–85) were present but undetected, highlighting the need for ongoing postborder surveillance to detect new incursions. We cannot ascribe these undetected species to a specific introduction period or sector, but our results indicate that about 90% of pathogens have been detected, meaning our overall findings should be robust.

In conclusion, we provide the first detailed analysis of plant pathogen arrival rates through time, accounting for variation in survey effort in a country that invests heavily in border biosecurity. Our analysis revealed that for the first half of the 20th century, the rate at which plant pathogens arrived and established on economically important plant species in New Zealand increased in concert with increasing import trade volume but was not linked to passenger arrivals. For crop and pasture species, pathogen arrival rates started diverging from imports around the 1960–1970s, coinciding with a greater biosecurity effort designed to limit pest and pathogen arrivals in the agricultural sector. Biosecurity measures appear to have been less effective in preventing pest and pathogen arrivals in the forestry and fruit tree sectors until recently, which may explain why pathogen arrival rates for woody species have continued to increase in recent decades. Our findings provide the first evidence, to our knowledge, that targeted investment in biosecurity may be effective in reducing pathogen arrival, despite increasing trade, and limiting the establishment of microorganisms but highlight the importance of sustained surveillance due to the significant risk, posed by increasing levels of trade, for unwanted introductions in the absence of effective biosecurity measures.

## Materials and methods

### Plant–fungal associations in New Zealand

We compiled a database of observed host–fungal (senso lato) associations in New Zealand recorded between 1847 and 2012. Each record comprised an observation of a fungus and its associated host plant and the year of observation. The data are stored in the NZFungi2 database (Landcare Research; http://nzfungi2.landcareresearch.co.nz/; [44]) and comprise essentially all known host–fungal records from New Zealand. The New Zealand economy’s historical reliance on primary production has meant there have been repeated systematic surveys of the diseases associated with agriculture, horticulture, and forestry [57–59] carried out by government agencies tasked with the diagnosis and surveillance of plant diseases [60]. Consequently, while the database includes native host plants, most records are of fungal taxa associated with introduced, economically important hosts. For well-surveyed host plants, there are typically multiple records of a given host–fungal association (mean of 4.9 records per association for hosts with more than 50 records), reflecting observations at different times in different parts of the country as part of surveillance efforts.

We standardised fungal and plant taxonomic names and removed duplicate entries and invalid names from the database [61]. We pooled all records at the species level and excluded border intercepts and hybrids, with the exception of well-sampled commercial hybrid plants (i.e., *Fragaria × ananassa*, *Cupressus × leylandii*, *Malus × domestica*). Database processing was performed in R [62].

### Plant–pathogen associations

We filtered the database to include only introduced host plants associated with four primary production sectors—crops (46 species), fruit trees (30 species), commercial forestry (42 species), and pastures (13 species sensu [63])—and included only species with at least 10 records (See S2 Table). We included all nonnative pathogens (including fungi, oomycetes, and plasmodiophorids) for which the first record for the pathogen in New Zealand was on one of the selected host species. Pathogen status was determined by expert opinion (A. Stewart, P. Johnston), and the nonnative status of pathogens in New Zealand was drawn from NZFungi2 [44,61].

### Modelling pathogen arrival over time

The records of host–pathogen associations in our data allow us to identify the year in which a particular pathogen was first discovered on an introduced host plant in New Zealand and thus to document the rate at which pathogens accumulated on host plants over time. The observed rate of pathogen accumulation, however, results from both an arrival and discovery process [42,43], and we developed a statistical model to separate these two processes, drawing on the approach described by Belmaker and colleagues in 2009 [64]. To ensure we had adequate sample sizes to quantify changes in pathogen arrival rates over time, we pooled host plant species and examined the accumulation of new pathogens across all species and across species in each of the four production sectors (crop, forestry, fruit tree, and pasture). Because our interest was in the effectiveness of border biosecurity measures, we did not examine the spread of pathogens from one host to another postborder.

For each group of host plant species in each year *t*, we have data on the number of new pathogen species discovered on those hosts, *D*_*t*_. We would like to know the number of pathogens that actually arrived and colonised those hosts in each year, *A*_*t*_, and the mean arrival rate, *μ*_*t*_, but we cannot observe these outcomes directly, and we have to estimate them from data on the number of discoveries in each year, *D*_*t*_, and sampling effort, measured here as the total number of host–pathogen records recorded in each year on a host plant group, *N*_*t*_ (see Fig 1).

To do this, we assume that for each host–pathogen record in a given year, there is a probability, *p*_*t*_, that the record is the discovery of a new pathogen species. If there are *N*_*t*_ host–pathogen records in year *t*, then the number of new pathogens discovered in that year can be modelled as a draw from a binomial distribution with probability *p*_*t*_:

$$D_{t}\sim \text{Binomial}\;\left(p_{t},N_{t}\right)$$

The actual number of pathogen species arriving and colonising a group of host species in a given year, *A*_*t*_, is unknown, and we model it as a random variable drawn from a negative binomial distribution with mean arrival rate *μ*_*t*_ and dispersion parameter *r*. We specified a negative binomial distribution to allow for the possibility that the number of pathogens arriving in each year might exhibit greater variation than would be expected under a Poisson distribution, which is a common distribution for modelling number of events per unit time:

$$A_{t}\sim \text{Negative}\;\text{Binomial}\;\left(\mu_{t},r\right)$$

The first year (*t* = 1) was set to the first year a host–pathogen association was recorded for a given group of host plants. Many host plant species, however, would have been present in New Zealand prior to the first record in the database and may have had pathogens with them when they arrived and been accumulating new pathogens since arrival. To allow for this, we included a term *A*_0_ to represent the number of pathogens already present on host species when the first host–pathogen association was recorded in the database. The number of pathogen species available to be discovered in year *t* is then equal to the total number of pathogen species that had arrived by the end of that year ($A_{0}+\sum_{t=1}^{t}A_{t}$) minus the total number of pathogen species that had been discovered at the start of that year ($\sum_{t=1}^{t-1}D_{t}$). We can estimate the probability, *p*_*t*_, that a host–pathogen observation in year *t* was a newly discovered pathogen as the number of undiscovered pathogen species in year *t* divided by the total number of pathogen species that have arrived:

$$p_{t}=\left(\frac{A_{0}+\sum_{t=1}^{t}A_{t}-\sum_{t=1}^{t-1}D_{t}}{A_{0}+\sum_{t=1}^{t}A_{t}}\right)$$

Finally, we modelled the mean rate of pathogen arrival, *μ*_*t*_, as a function of time *t*. We fitted a semiparametric regression model using penalised splines to allow the shape of the curve describing arrival rate through time to be determined by the data. We followed the method from Crainceanu and colleagues in 2005 [65] and fitted a low-rank thin-plate spline of the form:

$$\mu_{t}=\beta_{0}+\beta_{1}t+\overset{K}{\underset{k=1}{\sum }}b_{k}|t-\kappa_{k}{|}^{3},$$

where *t* is time; *β*_0_, *β*_1_, and *b*_*k*_ are regression coefficients; and *κ*_1_ < *κ*_2_ …< *κ*_*K*_ are fixed knots that determine the flexibility of the spline. We chose *K* = 5 knots and specified the location of each knot at the quantiles of *t* corresponding to probability *k* / (*K* + 1), thus ensuring the knots were evenly spaced over time. The above spline can be expressed in the form of a linear mixed-effects model and can therefore be fitted using software for mixed or hierarchical models [65].

We fitted the above model to the data in a Bayesian framework using Markov Chain Monte Carlo (MCMC) simulation implemented in JAGS called from R through the jagsUI package [66]. For *A*_*0*_, we specified a uniform prior in the range 0 to the total number of pathogens recorded; for *r*, the dispersion parameter of the negative binomial distribution, we specified a uniform prior in the range 0–500; for the parameters *β*_0_ and *β*_1_, we specified a flat normal prior with mean 0 and standard deviation 10,000. To avoid overfitting, we penalised the parameters *b*_*k*_ by modelling these as drawn from a normal distribution with mean 0 and variance estimated from the data [65]. We ran our models with 3 chains using the function autojags to ensure the chains converged. We first ran the chains for 10,000 iterations with a 5,000-iteration burn-in. At the end of this run, the autojags function assessed the chains for convergence, defined as the Gelman-Rubin statistic being less than 1.1 for all sampled parameters [67]. If the Gelman-Rubin statistic was greater than 1.1, a further 5,000 iterations were run, and this was repeated until the chains had converged.

### Trade and passenger data

Data on overall trade volume came from the tonnage of international cargo unloaded at New Zealand ports, obtained from combining Overseas Trade Statistics data 1923–1988 [68] with more recent data from StatsNZ Infoshare (http://www.stats.govt.nz/infoshare), in which data were only available from 1989–2017. We also evaluated data on the value of trade imports to New Zealand for the period 1914–2011, which were available from StatsNZ Infoshare as well. Raw data on import values were available for the period 1841–2011, but we used the Consumer Price Index (CPI) to inflation adjust these to NZ$ in 2012, and CPI data were only available from 1914 onward (StatsNZ Infoshare: http://www.stats.govt.nz/infoshare). Cargo tonnage and the value of trade imports were highly correlated (r^2^ = 0.92; S1 Fig). We used volume as it is more likely to reflect the ‘size’ of the potential pathogen pathway by measuring the quantity rather than value of material entering the country. We used international passenger arrival count data from 1900 to present (includes both international visitors and returning residents), also available through StatsNZ Infoshare. For import trade and passenger arrivals, we used a loess smoothing function to capture the trend in arrival rate over time.

Sector-specific trade data for the period 1960–2012 were obtained from the UN Food and Agriculture Organization (FAO). The FAOSTAT dataset (http://www.fao.org/faostat/en/#data/TP) catalogues imports of plant-based commodities in different categories. We selected those categories likely to be potential sources of plant pathogens (See S3 Table): Seeds, dry or fresh, hulled or unhulled, were included as long as they have not been roasted or milled, and only fresh, unprocessed fruit was included, with all dry or preserved fruit excluded. In addition, we determined the plant families associated with each plant commodity category (based on the species included in the commodity category) and excluded any commodity categories where the plant family was not present in our host plant data, since taxonomic affiliation of host plants is a key indicator of susceptibility and spread of pathogens among plants [61]. Import volumes for pasture products were very low, but several crops are in the same family as most pasture species (grasses and legumes). As such, pasture imports were coupled with these selected crop species as a measure of potential sources of imported pathogens for this sector. Forestry import volume data were obtained from the FAO forestry site (http://www.fao.org/faostat/en/#data/FO). Commodity item classes for forestry encompass species with broad taxonomic affiliations, so we excluded only nonconiferous tropical wood products, as there are no economically important nonconiferous tropical species grown in New Zealand, and this category made up less than 1% of forestry imports.

## Supporting information

S1 Table Number of detected fungal pathogens in New Zealand compared to those observed globally for the focal host species with the 10 most pathogens in New Zealand. (DOCX)

S2 Table List of focal host plant species and family affiliation for each production sector. (DOCX)

S3 Table FAO commodity categories and plant family affiliation used to determine sector-specific imports. FAO, Food and Agriculture Organization.
(DOCX)

S1 Fig Correlations between variables representing trade and passenger arrivals to New Zealand. (DOCX)

## Abbreviations

CPI: consumer price index
FAO: Food and Agriculture Organization
GDP: gross domestic product
MCMC: Markov Chain Monte Carlo
OECD: Organisation for Economic Co-operation and Development
