# Supplementary material for: Import volumes and biosecurity interventions shape the arrival rate of fungal pathogens
Source: PLoS Biol. 2018 May 31;16(5):e2006025. doi: 10.1371/journal.pbio.2006025 (PMC5978781; doi:10.1371/journal.pbio.2006025)
Supplement: S1 Table — (DOCX) [file pbio.2006025.s001.docx]

# Supplementary Information

S1 Table

Number of detected fungal pathogens in New Zealand compared to those observed globally for the focal host species with the ten most pathogens in New Zealand. For each host species, data include the number of fungal pathogens identified in the NZ Fungi2 database, the total number of fungal pathogens listed in the CABI database, the total number of unique fungal pathogens in the two databases combined, the number of CABI-listed fungal pathogens in New Zealand, the percent of all CABI-listed fungal pathogens currently found in New Zealand, and the percent of fungal pathogens in either database that are currently in New Zealand.

| Host Species | Host Sector | Pathogens in NZ | CABI-listed Pathogens | Total Pathogens | CABI Pathogens in NZ | % CABI Pathogens in NZ | % Total Pathogens in NZ |
| --- | --- | --- | --- | --- | --- | --- | --- |
| *Actinidia deliciosa* | Fruit trees | 32 | 19 | 43 | 8 | 42% | 74% |
| *Brassica oleracea* | Crops | 32 | 16 | 40 | 8 | 50% | 80% |
| *Hordeum vulgare* | Crops | 41 | 77 | 93 | 27 | 35% | 44% |
| *Lolium perenne* | Pasture | 50 | 23 | 60 | 13 | 57% | 83% |
| *Malus domestica* | Fruit trees | 45 | 88 | 112 | 22 | 25% | 40% |
| *Medicago sativa* | Pasture | 40 | 58 | 76 | 23 | 40% | 53% |
| *Pinus radiata* | Forestry | 52 | 27 | 70 | 9 | 33% | 74% |
| *Solanum lycopersicum* | Crops | 48 | 103 | 124 | 30 | 29% | 39% |
| *Triticum aestivum* | Crops | 44 | 94 | 110 | 29 | 31% | 40% |
| *Zea mays* | Crops | 33 | 101 | 117 | 18 | 18% | 28% |
