# Supplementary material for: Import volumes and biosecurity interventions shape the arrival rate of fungal pathogens
Source: PLoS Biol. 2018 May 31;16(5):e2006025. doi: 10.1371/journal.pbio.2006025 (PMC5978781; doi:10.1371/journal.pbio.2006025)

S2 Table

List of focal host plant species and family affiliation for each production sector.

| Sector | Host Species | Family |
| --- | --- | --- |
| Crop | *Beta vulgaris* | Amaranthaceae |
| Crop | *Allium cepa* | Amaryllidaceae |
| Crop | *Allium fistulosum* | Amaryllidaceae |
| Crop | *Allium sativum* | Amaryllidaceae |
| Crop | *Allium schoenoprasum* | Amaryllidaceae |
| Crop | *Apium graveolens* | Apiaceae |
| Crop | *Daucus carota* | Apiaceae |
| Crop | *Foeniculum vulgare* | Apiaceae |
| Crop | *Pastinaca sativa* | Apiaceae |
| Crop | *Petroselinum crispum* | Apiaceae |
| Crop | *Asparagus officinalis* | Asparagaceae |
| Crop | *Cynara scolymus* | Asteraceae |
| Crop | *Helianthus annuus* | Asteraceae |
| Crop | *Lactuca sativa* | Asteraceae |
| Crop | *Armoracia rusticana* | Brassicaceae |
| Crop | *Brassica napus* | Brassicaceae |
| Crop | *Brassica oleracea* | Brassicaceae |
| Crop | *Brassica rapa* | Brassicaceae |
| Crop | *Raphanus sativus* | Brassicaceae |
| Crop | *Humulus lupulus* | Cannabaceae |
| Crop | *Ipomoea batatas* | Convolvulaceae |
| Crop | *Citrullus lanatus* | Cucurbitaceae |
| Crop | *Cucumis melo* | Cucurbitaceae |
| Crop | *Cucumis sativus* | Cucurbitaceae |
| Crop | *Cucurbita maxima* | Cucurbitaceae |
| Crop | *Cucurbita moschata* | Cucurbitaceae |
| Crop | *Cucurbita pepo* | Cucurbitaceae |
| Crop | *Arachis hypogaea* | Fabaceae |
| Crop | *Glycine max* | Fabaceae |
| Crop | *Phaseolus coccineus* | Fabaceae |
| Crop | *Phaseolus vulgaris* | Fabaceae |
| Crop | *Pisum sativum* | Fabaceae |
| Crop | *Vicia faba* | Fabaceae |
| Crop | *Mentha spicata* | Lamiaceae |
| Crop | *Linum usitatissimum* | Linaceae |
| Crop | *Avena sativa* | Poaceae |
| Crop | *Hordeum vulgare* | Poaceae |
| Crop | *Secale cereale* | Poaceae |
| Crop | *Triticum aestivum* | Poaceae |
| Crop | *Zea mays* | Poaceae |
| Crop | *Capsicum annuum* | Solanaceae |
| Crop | *Nicotiana tabacum* | Solanaceae |
| Crop | *Physalis peruviana* | Solanaceae |
| Crop | *Solanum lycopersicum* | Solanaceae |
| Crop | *Solanum melongena* | Solanaceae |
| Crop | *Solanum tuberosum* | Solanaceae |
| Forest | *Sequoia sempervirens* | Cupressaceae |
| Forestry | *Betula pendula* | Betulaceae |
| Forestry | *Chamaecyparis lawsoniana* | Cupressaceae |
| Forestry | *Cryptomeria japonica* | Cupressaceae |
| Forestry | *Cupressus lusitanica* | Cupressaceae |
| Forestry | *Thuja plicata* | Cupressaceae |
| Forestry | *Acacia baileyana* | Fabaceae |
| Forestry | *Acacia dealbata* | Fabaceae |
| Forestry | *Acacia decurrens* | Fabaceae |
| Forestry | *Acacia longifolia* | Fabaceae |
| Forestry | *Acacia mearnsii* | Fabaceae |
| Forestry | *Quercus robur* | Fagaceae |
| Forestry | *Juglans regia* | Juglandaceae |
| Forestry | *Eucalyptus botryoides* | Myrtaceae |
| Forestry | *Eucalyptus cinerea* | Myrtaceae |
| Forestry | *Eucalyptus delegatensis* | Myrtaceae |
| Forestry | *Eucalyptus fastigata* | Myrtaceae |
| Forestry | *Eucalyptus fraxinoides* | Myrtaceae |
| Forestry | *Eucalyptus globulus* | Myrtaceae |
| Forestry | *Eucalyptus leucoxylon* | Myrtaceae |
| Forestry | *Eucalyptus nitens* | Myrtaceae |
| Forestry | *Eucalyptus ovata* | Myrtaceae |
| Forestry | *Eucalyptus regnans* | Myrtaceae |
| Forestry | *Eucalyptus saligna* | Myrtaceae |
| Forestry | *Larix decidua* | Pinaceae |
| Forestry | *Larix kaempferi* | Pinaceae |
| Forestry | *Pinus canariensis* | Pinaceae |
| Forestry | *Pinus contorta* | Pinaceae |
| Forestry | *Pinus elliottii* | Pinaceae |
| Forestry | *Pinus muricata* | Pinaceae |
| Forestry | *Pinus nigra* | Pinaceae |
| Forestry | *Pinus pinaster* | Pinaceae |
| Forestry | *Pinus ponderosa* | Pinaceae |
| Forestry | *Pinus radiata* | Pinaceae |
| Forestry | *Pinus strobus* | Pinaceae |
| Forestry | *Pinus taeda* | Pinaceae |
| Forestry | *Pseudotsuga menziesii* | Pinaceae |
| Forestry | *Populus alba* | Salicaceae |
| Forestry | *Populus nigra* | Salicaceae |
| Forestry | *Populus trichocarpa* | Salicaceae |
| Forestry | *Populus yunnanensis* | Salicaceae |
| Forestry | *Salix babylonica* | Salicaceae |
| Horticulture | *Actinidia chinensis* | Actinidiaceae |
| Horticulture | *Actinidia deliciosa* | Actinidiaceae |
| Horticulture | *Annona cherimola* | Annonaceae |
| Horticulture | *Carica papaya* | Caricaceae |
| Horticulture | *Vasconcellea pubescens* | Caricaceae |
| Horticulture | *Diospyros kaki* | Ebenaceae |
| Horticulture | *Vaccinium corymbosum* | Ericaceae |
| Horticulture | *Ribes nigrum* | Grossulariaceae |
| Horticulture | *Ribes uva-crispa* | Grossulariaceae |
| Horticulture | *Persea americana* | Lauraceae |
| Horticulture | *Ficus carica* | Moraceae |
| Horticulture | *Acca sellowiana* | Myrtaceae |
| Horticulture | *Olea europaea* | Oleaceae |
| Horticulture | *Passiflora edulis* | Passifloraceae |
| Horticulture | *Cydonia oblonga* | Rosaceae |
| Horticulture | *Eriobotrya japonica* | Rosaceae |
| Horticulture | *Malus domestica* | Rosaceae |
| Horticulture | *Prunus armeniaca* | Rosaceae |
| Horticulture | *Prunus avium* | Rosaceae |
| Horticulture | *Prunus dulcis* | Rosaceae |
| Horticulture | *Prunus persica* | Rosaceae |
| Horticulture | *Pyrus communis* | Rosaceae |
| Horticulture | *Pyrus pyrifolia* | Rosaceae |
| Horticulture | *Rubus idaeus* | Rosaceae |
| Horticulture | *Rubus ursinus* | Rosaceae |
| Horticulture | *Citrus limon* | Rutaceae |
| Horticulture | *Citrus reticulata* | Rutaceae |
| Horticulture | *Citrus sinensis* | Rutaceae |
| Horticulture | *Solanum betaceum* | Solanaceae |
| Horticulture | *Vitis vinifera* | Vitaceae |
| Pasture | *Lotus corniculatus* | Fabaceae |
| Pasture | *Lotus pedunculatus* | Fabaceae |
| Pasture | *Medicago sativa* | Fabaceae |
| Pasture | *Trifolium pratense* | Fabaceae |
| Pasture | *Trifolium repens* | Fabaceae |
| Pasture | *Trifolium subterraneum* | Fabaceae |
| Pasture | *Plantago lanceolata* | Plantaginaceae |
| Pasture | *Dactylis glomerata* | Poaceae |
| Pasture | *Festuca arundinacea* | Poaceae |
| Pasture | *Holcus lanatus* | Poaceae |
| Pasture | *Lolium multiflorum* | Poaceae |
| Pasture | *Lolium perenne* | Poaceae |
| Pasture | *Phleum pratense* | Poaceae |

Figure S1

Correlations between variables representing trade and passenger arrivals to New Zealand. The three metrics include import value, cargo volume, and passenger arrivals with data obtained mainly from NZ Infoshare (*Materials and Methods*)*.* Import value and cargo volume were linearly related (p <0.001) and highly correlated (r^2^= 0.92). Passenger arrivals to New Zealand were not well correlated either trade metric.


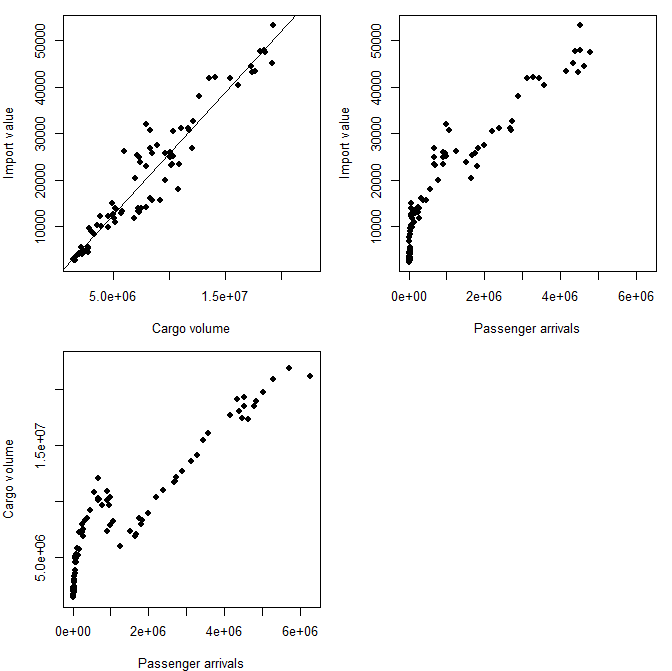

Supplement: S2 Table — (DOCX) [file pbio.2006025.s002.docx]
