# Supplementary material for: Import volumes and biosecurity interventions shape the arrival rate of fungal pathogens
Source: PLoS Biol. 2018 May 31;16(5):e2006025. doi: 10.1371/journal.pbio.2006025 (PMC5978781; doi:10.1371/journal.pbio.2006025)
Supplement: S3 Table — FAO, Food and Agriculture Organization. (DOCX) [file pbio.2006025.s003.docx]

S3 Table

FAO commodity categories and plant family affiliation used to determine sector-specific imports.

| **Item** | **Family** | **Sector** |
| --- | --- | --- |
| Spinach | Amaranthaceae | crop |
| Sugar beet | Amaranthaceae | crop |
| Garlic | Amaryllidaceae | crop |
| Leeks, other alliaceous vegetables | Amaryllidaceae | crop |
| Onions, shallots, green | Amaryllidaceae | crop |
| Anise, badian, fennel, coriander | Apiaceae | crop |
| Carrots and turnips | Apiaceae & Brassicaceae | crop |
| Asparagus | Asparagaceae | crop |
| Artichokes | Asteraceae | crop |
| Lettuce and chicory | Asteraceae | crop |
| Sunflower seed | Asteraceae | crop |
| Cabbages and other brassicas | Brassicaceae | crop |
| Cauliflowers and broccoli | Brassicaceae | crop |
| Mustard seed | Brassicaceae | crop |
| Rapeseed | Brassicaceae | crop |
| Hops | Cannabaceae | crop |
| Sweet potatoes | Convolvulaceae | crop |
| Melons, other (inc.cantaloupes) | Cucurbitaceae | crop |
| Cucumbers and gherkins | Cucurbitaceae | crop |
| Pumpkins, squash and gourds | Cucurbitaceae | crop |
| Watermelons | Cucurbitaceae | crop |
| Linseed | Linaceae | crop |
| Vegetables, fresh nes | List includes multiple focal crop families | crop |
| Buckwheat | Polygonaceae | crop |
| Strawberries | Rosaceae | crop |
| Chillies and peppers, green | Solanaceae | crop |
| Eggplants (aubergines) | Solanaceae | crop |
| Potatoes | Solanaceae | crop |
| Tomatoes | Solanaceae | crop |
| Beans, green | Fabaceae | crop, pasture |
| Chick peas | Fabaceae | crop, pasture |
| Groundnuts, shelled | Fabaceae | crop, pasture |
| Lentils | Fabaceae | crop, pasture |
| Peas, green | Fabaceae | crop, pasture |
| Soybeans | Fabaceae | crop, pasture |
| Barley | Poaceae | crop, pasture |
| Bran, maize | Poaceae | crop, pasture |
| Bran, wheat | Poaceae | crop, pasture |
| Canary seed | Poaceae | crop, pasture |
| Maize | Poaceae | crop, pasture |
| Millet | Poaceae | crop, pasture |
| Oats | Poaceae | crop, pasture |
| Rice - total (Rice milled equivalent) | Poaceae | crop, pasture |
| Rye | Poaceae | crop, pasture |
| Sorghum | Poaceae | crop, pasture |
| Wheat | Poaceae | crop, pasture |
| Alfalfa meal and pellets | Fabaceae | pasture |
| Straw husks | Poaceae | pasture |
| Forage products | Undefined; "plants grown specifically for animal feed" | pasture |
| Kiwi fruit | Actinidiaceae | fruit trees |
| Papayas | Caricaceae | fruit trees |
| Blueberries | Ericaceae | fruit trees |
| Chestnut | Fagaceae | fruit trees |
| Currants | Grossulariaceae | fruit trees |
| Walnuts, shelled | Juglandaceae | fruit trees |
| Walnuts, with shell | Juglandaceae | fruit trees |
| Avocados | Lauraceae | fruit trees |
| Fruit, fresh nes | List includes multiple focal fruit tree families | fruit trees |
| Fruit, tropical fresh nes | List includes multiple focal fruit tree families | fruit trees |
| Figs | Moraceae | fruit trees |
| Olives | Oleaceae | fruit trees |
| Almonds shelled | Rosaceae | fruit trees |
| Apples | Rosaceae | fruit trees |
| Apricots | Rosaceae | fruit trees |
| Cherries | Rosaceae | fruit trees |
| Peaches and nectarines | Rosaceae | fruit trees |
| Pears | Rosaceae | fruit trees |
| Plums and sloes | Rosaceae | fruit trees |
| Grapefruit (inc. pomelos) | Rutaceae | fruit trees |
| Lemons and limes | Rutaceae | fruit trees |
| Oranges | Rutaceae | fruit trees |
| Tangerines, mandarins, clementines, satsumas | Rutaceae | fruit trees |
| Grapes | Vitaceae | fruit trees |
| Raisins | Vitaceae | fruit trees |
