# Supplementary material for: Import volumes and biosecurity interventions shape the arrival rate of fungal pathogens
Source: PLoS Biol. 2018 May 31;16(5):e2006025. doi: 10.1371/journal.pbio.2006025 (PMC5978781; doi:10.1371/journal.pbio.2006025)

S1 Fig

Correlations between variables representing trade and passenger arrivals to New Zealand. The three metrics include import value, cargo volume, and passenger arrivals with data obtained mainly from NZ Infoshare (*Materials and Methods*)*.* Import value and cargo volume were linearly related (p <0.001) and highly correlated (r^2^= 0.92). Passenger arrivals to New Zealand were not well correlated either trade metric.


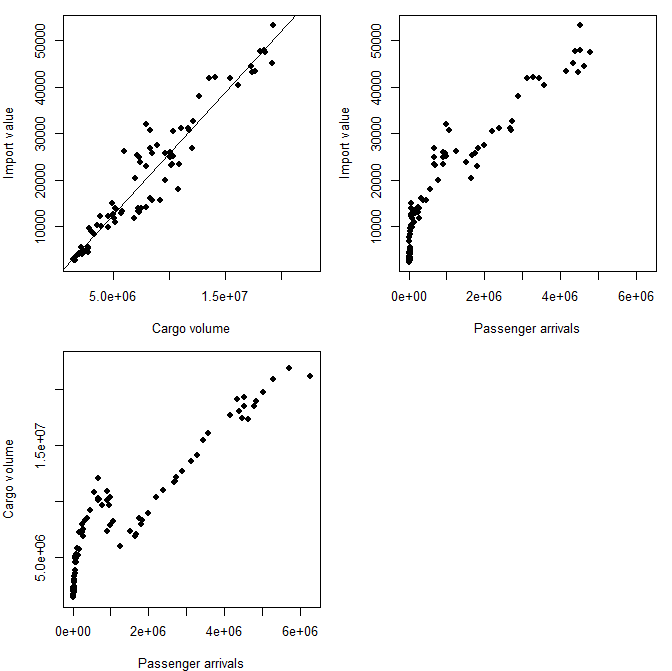

Supplement: S1 Fig — (DOCX) [file pbio.2006025.s004.docx]
